# An Essay on the Morbid Effects of Diseased Teeth upon the Human System

**Published:** 1844-06

**Authors:** W. W. H. Thackston

**Affiliations:** Fellow of the American Society of Dental Surgeons, of the Dental Society of Virginia, &c. &c.


### ARTICLE II.

*An Essay on the Morbid Effects of Diseased Teeth upon the Human System.* Read before the Virginia Society of Dental Surgeons, at their First Annual Meeting, October 11th, 1843, by W. W. H. THACKSTON, D. D. S., Fellow of the American Society of Dental Surgeons, of the Dental Society of Virginia, &c. &c. Published by order of the Society.

*Gentlemen of the Virginia Society of Dental Surgeons:*

HAVING been appointed to read an essay or dissertation, on some subject appertaining to the theory or practice of Dental Surgery, we have chosen the morbid effects of diseased teeth upon the human system, and the importance of their prompt removal, as a subject, perhaps, as well suited to the time and occasion, as any other upon which we could fix our attention.

When we remember the very intimate and extensive nervous connection which the dental apparatus sustains to the other parts of the system,—the various and multiplied sympathies incident to a diseased denture,—the exquisite local disturbance and pain, and the great constitutional derangement which such teeth occasion, it would appear almost a work of supererogation to say aught by way of impressing either practicien or patient with the urgent necessity and importance of the extraction of all teeth, or remains of teeth, which are found to be beyond the pale of restorative treatment.

This, however, we regret to say, is not the case, and notwithstanding the advanced and improved condition of our science, the superior facilities for cultivating an intimate acquaintance with correct pathological and therapeutical principles; which refer as directly to the diseases of the organs now under consideration, as to any of the maladies to which the human system is exposed; it is true, that a great majority of individuals will sooner incur the risk, and suffer the painful consequences of retaining such organs, than submit to the slight inconvenience and momentary suffering incident to their removal. But we are more pained to concede the fact, that there are now many, exercising the duties, and claiming the prerogatives and immunities of dental surgeons, who are too dark and ignorant to know or appreciate these truths, or who, knowing them, pusillanimously decline rendering that essential part of the service of the dental surgeon, because of the unpleasant nature of the operation, or a lack of confidence in their capacity for its performance, and that, too, often under the plea, that the presence of such organs will not only be harmless, but beneficial, in giving strength and support to the remaining healthy teeth, jaws, &c., and by awakening some of the prejudices and apprehensions, which every where exist with reference to the operation of extraction, they rarely fail to convince their patients, that however necessary and important this operation may be in some other instances, it is scarcely needful in their own.

From the foregoing considerations, we have been induced to make our selection, together with the hope that what we shall say may be of some avail, in correcting an error from which society has suffered much, and which has by no means enhanced the value and reputation of the profession to which you have devoted yourselves.

As the care of the teeth constitutes the chief purpose of our science, we will commence our essay by some reflections upon the injury inflicted on sound and healthy teeth, by the presence and proximity of diseased and dead organs of the same class. In the first place, we find such diseased organs most active and efficient agents in the propagation of disease to the remaining healthy teeth. And as there has been much diversity of opinion with reference to the pathology of caries, (the form of disease most common with the teeth,) we will here beg leave to digress for a moment, that we may make a few remarks upon this collateral subject.

This diversity of opinion has arisen from the conviction on the minds of several distinguished authors, that the immediate and exciting causes of this affection (caries) were *internal*, and the *development* of the disease *internal;* in other words, that decay always commenced in the interior structure of the tooth. Without going into a minute investigation of this question, we will simply observe, that after a most thorough and critical examination of the various agents and modes by which decay of the teeth is effected, their chemical characteristics and affinities, their physiological character and attributes; the question has been definitely settled, that the remote and predisposing causes of caries are *constitutional*, and the exciting, proximate and immediate causes *external*, its *development external*, and its character a chemical disorganization or decomposition of the dental structure, by the presence of an acrid and corrosive agent, which Dr. S. L. Mitchell, in a letter to Thos. Chas. Cope, M. D., of the University of Edinburg, states (from a series of experiments instituted for the purpose of determining the character of this agent) to be the *septic* or *nitrous* acid.

As we have previously observed, several authors of merited distinction, (and of this number perhaps none more so than Thos. Bell,) have stoutly maintained the doctrine of internal causes, and decay of the teeth; but subsequent investigations and experiments have demonstrated its fallacy, and we will only remark, in conclusion of this part of our subject, that the views here advanced are entertained by nearly all dental pathologists of the present day.

But to return from this digression; with the foregoing established facts before us, we shall be at no loss to perceive that the presence of diseased teeth is a direct and immediate cause of caries: directly, by retaining in contact with the sound organs the destructive agent; and indirectly, by vitiating and corrupting the mucous and salivary secretions; the effect of which, unless corrected by the most vigilant attention to the cleanliness of the parts involved, together with the removal of the diseased organs, will be the entire or partial loss of the teeth, with the unpleasant calamitous consequences incident thereto.

These considerations, though of vital importance, are not paramount to others, which, in their order, we shall briefly enumerate: first, in the train of consequences, will come odontalgia, with its exquisite and intense local suffering, frequently protracted for days and months, depriving its unhappy victims of ease and repose by night, until the whole system is unstrung and broken down by exhaustion; truly the Ayrshire bard has characterized it as the *"hell of all diseases."* Alveolar abscess is one of the most common terminations of odontalgia from decayed teeth; this being a formation of purulent matter about the *apices* of the fangs of the teeth, is retained until its quality becomes altered, and an acrid and corrosive character is imparted to it; the surrounding osseous structure is soon softened, and broken down sufficiently to afford an outlet for the pent up matter, which then developes itself in what is usually denominated a "gum boil," or fistulous ulcer, most frequently discharging its contents into the mouth, thus corrupting the breath, vitiating the secretions, and impairing the functional operations of the digestive apparatus; occasionally however, they develope themselves externally, and discharge through fistulous openings in the cheeks. Cases of this character, though not frequent, are by no means rare; we have, in our own practice, encountered two; in both instances, an inferior molaris and fang, which had been broken in an effort for its removal, were the exciting causes; in each case, the most disgusting fistulous ulcers were formed, which discharged, constantly, though slowly, an intolerably *fetid* and very *acrid* secretion.

The removal of the offending teeth, with, in one instance, a piece of *necrosed* bone, together with attention to the cleanliness of the parts, constituted the curative treatment; this course, we will here observe, is the only reliable one which can be adopted; no plan of treatment will succeed while the diseased teeth are suffered to remain; and we think we are safe in asserting, that in no instance will this plan fail, unless the state of the system be peculiarly unfavorable, or the case be of such long standing as that the edges of its duct or outlet have become callous; in the first instance such constitutional treatment as is indicated must be adopted, together with the local means which we have already suggested; in the latter, the removal of the hardened edges, with a narrow pointed bistoury, and drawing the wound together with adhesive straps, will generally effect a cure.

We will here, gentlemen, call your attention to the occurrence of *alveolar abscess, previous* to the moulting of the temporary or *deciduous* teeth; these teeth, it will be remembered, sustain not only the same nervous and vascular connection with the general system which the permanent ones do, but also an intimate *membranous* connection with the rudiments or pulps of the permanent set; and not only does this membranous union exist, but so crowded together are the fangs of the temporary, and pulps of the permanent sets, that in no case does any thing other than a thin spongy partition of bone separate them, and with the rudiments of the *bicuspides*, which succeed the temporary *molares*, this separation scarcely exists, if it does at all, the fangs of the latter completely enclosing them.

It may then readily be perceived, inasmuch as we know that from the imperfect organization of the temporary teeth, and from the action of external causes, that they are as liable, if not more so, than the succeeding denture to decay and ache, and that the same local and general effects are produced in the one instance as in the other; that the occurrence of abscess in this instance cannot be too much dreaded, and remedies cannot be too promptly employed to arrest their disastrous consequences. The permanent teeth being in their embryo state, and directly exposed to the action of the acrid and corrosive matter thus secreted, are, if not wholly destroyed, (which, by the way, frequently occurs,) so much impaired as never to receive that perfect organization and development so essential to their beauty, utility, and preservation.

The practical deductions which we would here make are, that every means should be called in requisition to prevent the development of abscess from a diseased temporary tooth,—as the application of soothing drops to the exposed nerve, local and general depletion, cooling aperients, pediluvia, &c. &c. But if abscess forms, in despite of all efforts to the contrary, the offending tooth should be *promptly* and *unhesitatingly* removed; it may here properly be remarked, that this class of teeth (the deciduous) should never be prematurely removed, (lest a contraction of the maxillary arch be the result,) unless for this, or some other necessity equally urgent; in the present instance extraction should be adopted as the lesser of two evils.

Scurvy of the gums, as it is generally called, though an affection more frequently *symptomatic* than *idiopathic*, is, if not developed by the irritation produced by the presence of diseased teeth, at least always aggravated by that cause; the same remark will also apply to all the morbid changes and conditions which the soft parts of the mouth undergo, as preternatural and spongy growths of the gums, the occurrence of *simple, cartilaginous, osteo sarcomatous, cancerous* and other *tumors.* The predisposition and various characteristics which these affections assume, are dependant upon a *specific constitutional diathesis;* but their exciting and developing causes are generally local, and in almost all instances where those tumors and other diseased actions are found in the mouth, the local cause is ascertained to be an impaired condition of the teeth; it is, however, proper to observe, that the mouth, in common with other parts of the system, may become the seat of those several forms of disease, *without* the intervention of the aforementioned excitant, such cases are, however, comparatively rare.

The various forms of disease incident to the *maxillary sinus* are more frequently produced by the secondary irritation propagated from diseased teeth, than any other cause whatsoever; but as our views upon this subject have been given in another place,* we will not here recapitulate them.

*Ozæna*, an ulcerous affection of the Schneiderian membrane, of a peculiarly unpleasant character, on account of its painful symptoms, and the intolerably fetid odor exhaled from it, is indebted, in many instances, for its existence, to an impaired denture; of this affection, Professor Harris has reported, in the Maryland Medical and Surgical Journal, a case accompanied with paroxysms of *neuralgia faciei,* which after resisting various forms of remedial treatment, including visits to several of our medicinal springs, was radically cured in a few weeks, by the extraction of a diseased *dens molaris.*

It not unfrequently happens that, through the medium of what is denominated sympathy, the teeth become most prolific sources of intense and agonizing pain; connected as they are by the union of the *pterygoid* branch of the *super maxillary,* with the first cervical ganglion of the *great sympathetic* nerve, a ready

* See Journal of Dental Science, vol. 2, p. 227.

medium of communication is established between them and other and remote parts of the system, and often are they (the teeth) the unsuspected cause of the most aggravated and painful symptoms developed in those parts; and that, too, without the least pain or indication that would lead to the detection of the primarily affected organ; and, as a natural consequence, the treatment usually employed can at best be rarely more than palliative, while, in many instances, it doubtless contributes to the suffering of the patient. As a case of this character, Dr. Rush, in his "Medical Enquiries," mentions an instance in which rheumatism of the hip joint was produced by a decayed tooth, and which, resisting the ordinary remedies, alone yielded to its extraction.

*Neuralgia* may also be mentioned as in many instances having its origin in a defective denture; it, however, exists most commonly as a constitutional disease, caused, according to McCullock and several other authors who have written upon the subject, by *miasmata, malarious exhalations, &c.* Mr. Bell is of opinion that this disease "may be divided into two distinct affections, distinct in their causes, and equally so in the effect which different remedies are found to exert upon them," the one produced by causes operating upon the system, and subject only to constitutional treatment,—the other dependant upon local causes, and, to almost the same extent, subject to local treatment. The distinguishing characteristics of those affections, according to Mr. Bell, (whose authority we consider, upon this subject, reliable,) is the regularity of the recurrence, &c. of the paroxysms in the constitutional form; while with the local affection the paroxysms are irregular in their recurrence, duration and degree of intensity.

This author details a number of cases of well marked *neuralgia*, of the local character, which, after the employment of constitutional remedies had failed to produce relief, or palliate the symptoms, were readily cured by the local treatment indicated. One of those cases we will transcribe, not because of any peculiarity which it possesses, for numbers of a similar character might be adduced, did we wish to swell our essay by their introduction.

"Mr. D., a gentleman aged about fifty, applied to me in consequence of a severe pain occurring in irregular paroxysms, first attacking the ear, then darting down the neck and shoulders, through the entire length of the arm, so as considerably to diminish the power of the hands; he had been for more than a year the subject of this affection, and had latterly consulted a physician of the highest standing, who, finding the medical treatment which he recommended had failed to produce the slightest relief, requested me to see him. I was informed that the second inferior *molaris* had been broken, in an attempt to extract it, about two years before, the roots of which were now remaining in the jaw, the anterior one having been partially thrust out of the alveolus, and lying obliquely upon the gum, the posterior one still remained firmly fixed in its socket, but evidently exerting much irritation in the surrounding parts, with increased pain on pressure, which assumed somewhat the character of the paroxysms which he had so long suffered. I therefore removed both roots, and had the satisfaction of hearing in a few weeks, that the complaint had entirely ceased."

Two cases of a similar character, in almost every respect, to the one above quoted, have come under our own observation, and another was mentioned to us a few days since by a professional acquaintance; in each instance, the removal of a diseased tooth caused an immediate subsidence of the paroxysms.

*Opthalmia, ear-ache* and *deafness* may be mentioned as common results of dental irritation; corroborative cases, in any number, might be here adduced, but as we do not wish to enlarge our paper with unnecessary matter, and as cases of this character are so common as to have come under the observation of almost every practitioner, we will pass on to the consideration of other diseased actions, induced by decayed and dead teeth.

*Hemicrania* is mentioned by Dr. Darwin, and observed by nearly every one of much experience, to be the result of dental irritation. Dr. D., in speaking of this affection, observes, "that it is attended with cold skin, and whatever may be the remote cause, the immediate one seems to be a want of stimulus, either of heat or distension, or some other unknown stimulus in the painful part, or in those with which it is associated. The membranes, in their natural state, are only irritable by distension, but in a diseased state they are sensible like muscular fibre;" hence, a diseased tooth may render the neighboring membranes sensible, and is, doubtless, frequently the cause of this disease." Of *sympathetic headache*, he observes, "where this affection is confined to a defined part on the *parietal* bone, or on one side, it is generally termed *clavis hystericus*, and is always, I believe, owing to a diseased *dens molaris*."

In addition to the above, we will detail a case which occurred in our own practice: Mrs. C., a lady of strumous habit, had long suffered severe paroxysms of *hemicrania*, accompanied, sometimes, with copious *emesis*, from which symptoms it was inferred that the *stomach* was disordered, and that the paroxysms were sympathetic, and a course of treatment predicated upon this supposition instituted, which, as the stomach was sympathetically affected, resulted in but little benefit. Having occasion to consult me with reference to the condition of her teeth, one of those paroxysms occurred while they were being examined. I found them generally affected with caries, some having lost their crowns entirely, leaving their spiculated remains protruding through the gums, which were spungy and turgid; one of the inferior molar teeth had been broken in an effort at its extraction, and was left with a living nerve exposed to the irritating effects of pressure, hot and cold drinks, particles of food, &c.

I at once concluded that the cause of her complaint had been overlooked or mistaken, and unhesitatingly urged the removal of every tooth and fang which could not be restored to perfect health. My suggestions were readily concurred in; and, with the removal of the offending organs, all traces of the malady disappeared.

*Epilepsy, catalepsy*, &c. are often developed by an impaired condition of the teeth. Of the former of those affections, Dr. Rush mentions the following case: "Some time in the year 1801, I was consulted by the father of a young gentleman of Baltimore, who had been for some time affected with *epilepsy*. I inquired into the state of his teeth, and was informed that several of them were decayed; I directed their removal, and advised the young man afterwards to lose a few ounces of blood, at any time when he felt the premonitory symptoms of a recurrence of his fits. He followed my advice, and in consequence, I had lately the pleasure of hearing that he was perfectly cured."

Of *catalepsy,* perhaps one of the most interesting cases on record, both on account of the singularity of the phenomena developed, the length of its duration, the varied treatment to which it was subjected, and its sudden and unexpected termination on the extraction of a diseased *inferior molaris,* has been reported by Dr. Black of Virginia; and, but for its great length, we would with great pleasure transcribe it; suffice it, however, to say, that it was one of those formidable and mysterious cases which almost every physician, at some time or other, encounters, and which, to his disappointment and mortification, baffles every form of treatment which he is most likely to institute; and which have for their cause, and curative indication, nothing more than a decayed tooth, and its removal.

*Phrenitis* may be enumerated as one of the affections somewhat rarely produced by disordered teeth, but that a malady so formidable is *ever* produced by this cause, is sufficient reason that the fact should be noted and dwelt upon, that the danger of retaining such teeth may be properly estimated. Of this affection, from dental irritation, a most interesting and conclusive case is given in the Journal and Library of Dental Science, vol. 2, page 141, by J. E. Snodgrass, M. D. The considerable length of the article will prevent our presenting it in detail, but the material points we shall give you:

The subject, (who was the father of Dr. S.,) from imprudent exposure, suffered an *odontalgic* attack, in which several of his teeth participated; the inflammatory action gradually extended itself, until the jaw and left ear became involved, attended with a drumming of the latter organ, dizziness of the head, which rapidly ran into inflammation of the brain, attended with *delirium* and *coma,* in which state he died a few hours after his attack. In the language of Dr. S., "I have not the least doubt that odontalgia was the indirect cause of my father's sudden mortality, the earache was doubtless *sympathetic,* while *phrenitis* was the *finale.*"

The various diseased actions, growing out of an impaired and deranged condition of the *digestive apparatus,* are connected in an intimate degree, with the condition of the dental organism, and we hazard nothing in saying, that though the affections in question are not always dependent upon a morbid condition of the teeth, they nevertheless often are, and by whatever other cause, they may be developed, the presence of such organs *never* fails, materially to aggravate them. In the process of assimilation, nothing is of greater importance, than that the food should be *perfectly* masticated, and that the *saliva* with which it is mixed, and by which it is fitted for deglutition and digestion, should be perfectly pure and healthy, otherwise the food is swallowed in an improper state, for with a diseased denture, it cannot be properly triturated; and the secretions being much vitiated, are both irritating to the mouth and nauseating to the stomach; the digestive organs thus having a two-fold duty to perform, the process is necessarily slow and painful, and as Prof. Harris justly remarks, "must necessarily weaken their powers and hasten their destruction." The immediate consequences of this imperfect mastication and insalivation, are *debility* to greater or less extent, sense of *oppression* in the region of the *stomach, nausea, sick headache,* &c., eventuating generally, in confirmed *dyspepsia.* We might cite a number of well authenticated cases of this character, but for reasons which we have previously stated, we shall, without farther remark, pass on to the last, in the catalogue of diseases, to which we shall particularly invite your attention on the present occasion, as having their origin in a diseased state of the teeth.

*Phthisis Pulmonalis.*—This appalling disease, in common with those to which we have already adverted, and others to which we might revert, were it compatible with the limits of our paper, is occasionally the melancholy consequence of the ignorance, or negligence of those who suffer their teeth to go to ruin, without an effort for their restoration, and who, from some silly apprehension, or ill-grounded prejudice, persist in retaining their irritating remains. We know that this declaration may startle some, nevertheless it is strictly true, and may be readily and rationally explained, by the extension of dental irritation, involving first the soft parts of the mouth, the *palate, tonsils, pharynx and glottis,* and lastly, the *trachea, bronchia,* &c. &c., and by the corruption of the air, which passes through the mouth during the process of respiration. But as facts form a more convincing species of evidence than even the most philosophical speculations, we will adduce cases of well marked pulmonary consumption; cases, which from their symptoms, and the reputation of those under whose notice they came, and by whom they were reported, *cannot* be mistaken or doubted. We will, however, first introduce a just and eloquent passage, which occurs in the work of Dr. Fitch, on the subject of dental diseases, &c. This author, in treating upon the constitutional effects of diseased teeth and gums, observes, "that nature has formed the lungs most delicate and sensible, and susceptible to the slightest injurious impressions. She has also, finely tempered the atmosphere for its safe and healthy reception in those delicate organs; but art, accident or disease, may render it impure, unfit for respiration, and cause it instead of harmonizing with the lungs in the most perfect manner, and giving to them, and the whole system, health and strength, to be a baneful influence, armed with pestilence, scattering the seeds of disease over the lungs, and pouring the streams of deadly poison through every vein of the system."

We find in the excellent work of the author just quoted, the following case: "In February, Dr. Samuel Jackson, of this city, (Philadelphia,) called and requested me to see Mrs. R., who resided in Tenth, above Walnut street, who, he said, was laboring under every symptom of pulmonary consumption, and also appeared to suffer greatly from a diseased state of her mouth. I accordingly called on Mrs. R., and found the following to be her symptoms: great emaciation, hectic fever, almost constant cough, nearly a total loss of voice, articulation extremely difficult, as if speaking through a trumpet. Dr. Jackson said, that in a practice of seven years in the hospitals, alms houses, and private practice, he had never seen a case where a person recovered from the symptoms under which Mrs. R. laboured." I need not say that the Dr's practice was most extensive. The following was the condition of Mrs. R's mouth; about two years before, she had the upper wisdom tooth of the left side plugged, the filling being pounded in with a *mallet* and punch, by a dentist of this city; the fangs of the tooth converged so as to form a cone, and in hammering in the filling the socket was much injured; a chronic inflammation took place, which passed back over the *palate*, *half arches*, and some distance down the *œsophagus,* also over the *glottis, epiglottis* and *larynx;* it then passed over the right side of the under jaw, and caused to inflame and slough away, all the sockets and teeth of the *inferior maxilla* but one, the left *dens sapientiæ,* which had not become affected when I was consulted. When I first saw Mrs. R., the process of inflammation, sloughing and gangrene, was at its height; extensive exfoliations of the jaw were taking place, and Dr. Jackson and myself concluded that the patient could not survive more than four weeks. The treatment of this case was as follows: I at once removed all of the teeth which were loose, and whose sockets were in a state of gangrene and exfoliation, likewise as much as possible of the necrosed bone, and directed a strong infusion of galls as a wash; in eighteen days the mouth became perfectly well, the amendment of her general health was surprisingly rapid; in five weeks she was able to take long walks in the streets, and in six months was restored to perfect health; nearly six years have since elapsed, and she continues in the enjoyment of excellent health."

This case, which differs in no material respect from many which have been reported, and which are almost daily occurring, speaks, in intonations which should sink deep into the minds of all who assume the responsibilities of the healing art, and especially those who have adopted that department which refers particularly to the diseases of the teeth and mouth.

It is sometimes objected against the extraction of such teeth as we have indicated, that they produce no pain, occasion no inconvenience; and why, in many instances exclaims the victim of his own folly, should I undergo the pain of having them removed? we would reply in the language of the great Dr. Rush, "when we consider how often the teeth, when decayed, are exposed to irritation from heat, and cold drinks, and aliments, from pressure, by mortification, and from the cold air, and how intricate the connexion of the mouth is with the whole system, I am disposed to believe that they are often the *unsuspected* cause of general, and particularly *nervous diseases.* When we add to the list of those diseases, the morbid effects of the acrid and putrid matter discharged from carious teeth, or from ulcers in the gums created by them, also the influence which both have in preventing perfect mastication, and the connection of that animal function with good health; I cannot help thinking, that our success in the treatment of all chronic diseases would be very much promoted by an examination of the condition of the teeth, and by advising their extraction in every instance in which they are found to be decayed. It is *not* necessary that they should be attended with pain in order to produce disease, for splinters, tumors, and other irritants *often* bring on *diseases* and *death*, when they give no pain, and are the unsuspected causes of them. This transition of sensation, and motion to parts remote from the place where impressions are made, appears in many instances, and seems to depend upon an original law of the economy."*

Dr. Koecker, in treating the same subject, holds the following language: "If these secondary local, and constitutional, affections, which are frequently mistaken for *primary* diseases, are treated *as such*, the symptomatic disorder will generally be much aggravated; those mistakes, are however, not surprising, when we consider the great difficulty there is in detecting the real and primary causes which are consequently, too frequently disregarded by the patient, and not unfrequently unperceived by the medical and surgical attendant. We cannot, therefore, too much bear in mind, that the *idiopathic* diseases of the mouth, even when in a very advanced state, *often* occasion the *least* pain in the part *primarily affected;* whereas the symptomatic disorders produced by them are *exceedingly* painful and alarming."†

We now beg leave to offer, in conclusion, a few reflections upon the facts which we have briefly presented. We find an impaired condition of the teeth, (we wish to be understood, as referring *only* to such teeth as cannot be restored to perfect health,) to be the occasion of much human suffering, the exciting agent of many human woes, of fearful and appalling maladies, the secret and destroying agent of many valuable lives; and all this injury and ruin affected by parts from which the unfortunate victims derive no benefit, which subserve no useful purpose, and which are retained only from an exaggerated idea of the pain experienced in their removal, or an unconsciousness of their imminent danger. Can the intelligent, then, be held blameless for persisting in this suicidal course? and will the physician or surgeon who fails to impress his

* Med. Enquiries, vol 1, page 201.

† Koecker's Principles of Dental Surgery.

patients with the urgent necessity of attending to this part of the preservation of their health, discharge the high and responsible duties which he owes to himself, his profession, and above all, to the individual who confides to him the care of his health, and the preservation of his life?

